# A “Round, Bruising Sort of Pain”: Autistic Girls’ Social Camouflaging in Inclusive High School Settings

**DOI:** 10.1007/s10803-024-06716-5

**Published:** 2025-01-10

**Authors:** Brittney L. Goscicki, Mattie E. Scoggins, Gabriela Herrera Espinosa, Robert M. Hodapp

**Affiliations:** Department of Special Education, Peabody College, Vanderbilt University, 110 Magnolia Circle, Nashville, TN 37212 USA

**Keywords:** Autism, Female, Camouflaging, Inclusion, School

## Abstract

Although autistic females often “camouflage” their autism, few studies examine the degree to which adolescent females demonstrate these behaviors in inclusive school settings. We examined: (a) the nature, extent, and underlying motivation of camouflaging in high school; (b) the extent to which autistic girls’ characteristics related to camouflaging settings, people, benefits, costs, and school supports; and (c) how girls’ open-ended descriptions agreed with closed-ended camouflaging ratings. Using quantitative and qualitative analyses, this study examined the extent, domains, costs, and benefits of autistic females’ school-based camouflaging. Thirty-one autistic female adolescents, all included in general education classrooms, answered rating and interview questions. Autistic females camouflaged most often in general education classrooms and with teachers and neurotypical peers that they did not know well; least often at home or with neurodivergent friends. Later age of diagnosis was associated with more camouflaging and camouflaging costs. Qualitative analyses revealed four themes: autistic identity; negative peer experiences; negative consequences of camouflaging; and value of neurodivergent friends. Some qualitative findings converged with quantitative findings, others diverged. Implications are discussed for research and practice for supporting autistic females in general education school settings.

## Introduction

Although autism has historically been much more often diagnosed in males than females, recent studies indicate that the male: female ratio may actually be 3:1 (Loomes et al., 2017). One potential reason for this discrepancy is that the criteria for many diagnostic tools may not “catch” autistic females. Instead, autistic females are more likely to get diagnosed if they have more “severe” symptoms (Dworzynski et al., 2012), and females with lower—versus higher—IQ’s may be more likely to receive an autism diagnosis (Van Wijngaarden-Cremers et al., 2014).

Beyond prevalence, females may present a different autistic phenotype (Hull et al., 2020). Girls, for example, often do not display the “typically autistic” interests like numbers or transportation. Instead, they may focus on people or animals (Lai et al., 2015). Autistic girls and boys might also attend to social and nonsocial stimuli differently, with eye-tracking studies showing that the visual attention of autistic girls is comparable to typically developing girls and different than autistic boys (Harrop et al., 2019). Compared to autistic boys, autistic girls also show greater social attention (Harrop et al., 2019), socially appropriate language skills (Hiller et al., 2014), and interests in friendships (Sedgewick et al., 2016).

These sex differences have implications in everyday contexts, including schools. Since the reauthorization of IDEA (2004) in the United States, students with disabilities are increasingly taught alongside typically developing peers, spending part of each day in general education classrooms (National Center for Education Statistics, 2023). Although inclusion is associated with a constellation of benefits for both students with and without disabilities (Wehmeyer & Kurth, 2021), simply placing students with disabilities into inclusive contexts does not guarantee belonging. If teachers and neurotypical students are not accepting of others with learning differences, students with disabilities can be placed in environments that leave them vulnerable to exclusion. Compared to neurotypical peers and other peers with disabilities, autistic teens experience peer victimization at a higher frequency (Greenlee et al., 2020).

But even here sex differences seem apparent. Studies of neurotypical adolescents indicate that girls are more likely to be involved in relational aggression compared to boys. Girls more often express anger and exclusion indirectly, with behaviors such as spreading rumors, gossiping, and eye-rolling (Card et al., 2008). Autistic girls might not even realize they are being bullied or get additionally confused over these more passively aggressive behaviors (Sedgewick et al., 2016). Further, for girls but not for boys, relational aggression predicts such anxiety symptoms as worrying about what others think, whether something was done poorly, or fainting when there is no reason (Greenlee et al., 2020). Autistic girls may also be more likely to internalize their reactions to such aggression, leading to greater distress and heightened anxiety (Greenlee et al., 2020).

Such sex differences must also be considered within the context of U.S. public schools (i.e., publicly funded schools that are free to students). Success in secondary school often entails complex academic and behavioral requirements. As students progress over the school years, they interact with different teachers and peers, and increasingly engage in group projects. Some autistic females find these situations difficult (Neal et al., 2016). Consider also the school’s physical environment, including such characteristics as spatial density, noise, lighting, and seating arrangements. In the traditional high school, students must transfer classes in noisy hallways and eat in crowded cafeterias; both can be difficult for those with sensory differences. Students may also be pulled out for related services such as speech-language therapy or for additional instructional support in separate resource rooms; in getting pulled out for services, girls might then “pick up” that they are different.

In research on autistic females’ school experiences, most studies have focused on friendships (e.g., Ryan et al., 2021; Sedgewick et al., 2019), peer victimization (e.g., Greenlee et al., 2020), general experiences with peers (e.g., Vine Foggo & Webster, 2017), and school overall (e.g., Tomlinson et al., 2022). However, little is known about camouflaging in schools. Defined as “the employment of specific behavioural and cognitive strategies by autistic people to adapt to or cope within the predominately non-autistic social world” (Cook et al., 2021, p. 1), camouflaging involves a discrepancy between (a) interpersonal behavioral presentation and (b) self-reported autistic traits and objectively measured social-cognitive abilities (Lai et al., 2016). For example, an autistic individual may mimic another person’s body language, force eye contact, and/or force themselves to engage in social situations (Hull et al., 2019). In a recent review, Goscicki (2023) found that, although researchers rarely directly asked about camouflaging in general education classes, autistic girls brought up camouflaging in over half of studies (53.8%; 14 studies). Camouflaging appears an important topic for female autistic students.

Camouflaging might also be particularly problematic for autistic teen girls. For females versus males, social pressures might be greater to display the “right” behaviors (Kreiser & White, 2014; Lai et al., 2015). Even with similar levels of autistic traits, autistic adolescent females (vs. males) have higher social reciprocity (e.g., reciprocal turn-taking, interaction, and flexibility) (Wood-Downie et al., 2021). Although males also camouflage (Pearson & Rose, 2021), the mental toll of camouflaging is likely greater for females. In one study, autistic adolescent males reported feeling more positively about camouflaging, whereas females reported more negative feelings and more often referenced their autistic identity (Bernardin et al., 2021b). Females were also more aware of why they camouflaged, citing a motive to avoid negative social experiences. In another study, Bernardin et al. (2021a) found that camouflaging was a predictor of depression, anxiety, and stress, but was particularly psychologically distressing (e.g., anxiety, depression) for females (with or without autism). Thus, mental health outcomes might vary by sex.

One potential explanation for these sex differences is that autistic females are more likely to have co-morbid internalizing disorders (e.g., depression, anxiety) with greater severity than autistic males (Kreiser & White, 2014), a sex-difference finding paralleled among typically developing youth and adults (Altemus et al., 2014). Further, adolescence marks a time during which social demands become more intense and complex. For example, friendships shift from playing together to talking together (Poulin & Chan, 2010). Further, in early school years, autistic girls are likely to be “mothered” in peer groups, whereas in secondary school they are bullied (Lai et al., 2015). Thus, in later school years, girls diagnosed with ASD might increasingly come across as “strange” to others and feel increasingly “different.”

A few preliminary studies also provide clues to the camouflaging behaviors of teen autistic girls in school. Observing elementary-aged children in playgrounds, Dean et al. (2017) found that autistic girls stayed close to peers, weaving in and out of activities, whereas autistic boys played alone, making ostracism easier to detect. In another study (Cook et al., 2018), parents of girls in mainstream (vs. specialized) settings more often talked about camouflaging. Examining autistic adolescents in both mainstream classes and in resource classes (in which students are exclusively taught alongside other students with special needs), Halsall et al. (2021) found that, in addition to camouflaging more in mainstream vs. resource-based classrooms, camouflaging attempts were largely unsuccessful, impacting girls’ sense of belonging and school relationships. Girls felt that they were neither “normal enough” to fit into inclusion nor “different enough” for specialist resource classrooms.

While these studies shed light on autistic girls’ camouflaging behaviors, critical issues remain. Within mainstreamed high schools, we do not yet know the extent to which camouflaging differs across other school settings (e.g., classroom versus cafeteria) and people (e.g., general education teachers, neurodiverse friends). The girls’ own perspectives are also generally missing, as few studies interview the girls themselves. To address these issues, this study addressed the following questions:

1. What is the nature, extent, and underlying motivation of camouflaging in high school among autistic females educated alongside neurotypical peers for most of the school day?
2. To what extent do autistic girls’ characteristics relate to camouflaging settings, people, benefits, costs, and school supports?
3. How do girls’ open-ended descriptions agree with closed-ended camouflaging ratings?

## Method

### Participants

Participants included 31 mother-daughter dyads. Mothers were mostly White, college educated, and married or in a domestic partnership. Daughters were also mostly White. The mean age of daughters was 15 years old, and most were in 9th grade. Most girls were diagnosed later in childhood (*m* = 10.90 years). Most girls had a co-occurring condition, most commonly anxiety. Over half took psychotropic medicines. Most girls had been included with neurotypical peers since preschool and spent all day in general education. Over half of girls had an Individualized Education Program (IEP). See Table 1.

**Table 1:** Demographic Characteristics of Participants

Characteristic		Mean (SD)	% (*n*)
**Mothers**			
Race	White		96.7% (29)
	Black		3.3% (1)
Education	High school		3.3% (1)
	Some college		13.3% (4)
	College degree		26.7% (8)
	Graduate school degree		56.7% (17)
Income	$20,001—$50,000		6.7% (2)
	$50,001—$80,000		23.3% (7)
	$80,001—$100,000		3.3% (1)
	More than $100,000		66.7% (20)
Employment status	Full time		60.0% (18)
	Part time		26.7% (8)
	Not employed outside home		13.3% (4)
Marital status	Married/domestic partnership		73.3% (22)
	Divorced/separated		23.3% (7)
	Widowed		3.3% (1)
Number typically developing children	0		43.3% (13)
	1		33.3% (10)
	2 +		23.4% (7)
Number autistic children	1		60.0% (18)
	2 +		40.0% (12)
Support group status	Yes		46.7% (14)
BAPQ		104.6 (26.0)	
Autistic	Yes		3.3% (1)
**Daughters**			
Race	White		93.5% (29)
	Black or multiracial		6.4% (2)
Age		15.45 (1.26)	
Age diagnosed		10.90 (4.92)	
Co-morbid conditions	None		25.8% (8)
	Anxiety		67.7% (21)
	Depression		35.5% (11)
	ADHD		45.2% (14)
	Other		22.6% (7)
Psychotropic medicine	Yes		58.1% (18)
SRS-2		162.4 (26.4)	
RCADs		48.6 (12.7)	
Aged Placed in inclusion	Preschool		77.4% (24)
	Elementary		12.9% (4)
	High school		9.7% (3)
Percent in inclusion	All Day		77.4% (24)
	Part of Day		22.6% (7)
IEP or 504 Plan	IEP		54.8% (17)
	504 Plan		22.6% (7)
	Neither		22.6% (7)
Grade	9		35.5% (11)
	10		29.0% (9)
	11		12.9% (4)
	12 +		22.6% (7)

Daughters were autistic female-identifying adolescents. Inclusion criteria were: (a) 13–18 years of age; (b) clinical diagnosis of autism; (c) a student in a public high school grades 9 through 12 (ages 13–18); (d) mainstreamed for at least one year for 80% of the school day. Daughters provided consent if they were 18 years old and assent if they were less than 18. Inclusion criteria for biological mothers were: (a) English-speaking and (b) lived in the same household as the adolescent or had joint custody. As one mother had two autistic daughters, that mother’s demographic information was included only once for data analyses.

### Recruitment

After receiving Institutional Review Board (IRB) approval, participants were recruited nationally. Emails and flyers were sent to state and local chapters (i.e., own board of directors, policies, and procedures) of various nonprofits, including the Arc, University Centers for Excellence in Developmental Disabilities, Autism TN, and graduates of the Volunteer Advocacy Project. Flyers were additionally posted on Facebook. We also recruited through the Simons Foundation Powering Autism Research for Knowledge (SPARK) database, a U.S.-based online research cohort (SPARK Consortium, 2018). Participants were encouraged to share the flyer with anyone they thought might be an interested, eligible participant.

Participants expressed interest by completing a consent to complete the prescreen followed by the prescreen itself. Although autism diagnoses were parent-reported, Daniels et al. (2012), examining the Interactive Autism Network (IAN) database, found that parental reports of diagnosis are valid. Eligible participants were contacted to arrange a Zoom meeting to discuss the consent process. Daughters and their mothers each received a $25 electronic Amazon gift card for participating.

### Survey Development

Following recommendations for designing accessible survey instruments for autistic individuals (Nicolaidis et al., 2020), efforts were made to limit jargon, confusing terms, and complex phrases. Response options were made as precise as possible and open-ended items allowed respondents to elaborate on experiences. We used identity-first language and refrained from classifying individuals by “functioning level.”

Survey and interview questions were then piloted with a member of the autism community outside the research team. This advocate provided feedback on individual questions, length of time to complete the survey, sufficiency of answer choices, and reasonableness of study compensation. Likert-style questions were further quantified based on frequency; for example, “always” was a behavior occurring five times a week at school, while “sometimes” occurred one to two times per week. We also clarified items: “anxious” was changed to “anxious (feeling nervous).” A revised draft survey was then sent to a faculty member and several special education graduate students, with further changes eliminating errors and increasing clarity.

### Data Collection

The study was comprised of two parts. In part one, mothers completed a 35–40-min web-based questionnaire about themselves and their daughters. In part two, autistic daughters and their mothers separately completed 30-min Zoom interviews about camouflaging in school. To ensure that items were accessible to all respondents, a researcher read aloud questions to participants and recorded close-ended answers. Participants saw the written form via screen sharing and daughters’ camouflaging questionnaires incorporated visual supports. For participants with complex communication needs, participants typed out their responses or used their regular communication system (e.g., one girl used a visual board). The background, Zoom questionnaires and interviews were recorded, submitted, and stored in REDCap (Harris et al., 2009). To transcribe open-ended responses, research assistants viewed recordings; fidelity checks from a research assistant ensured that questions were marked correctly and questions read aloud in the same order.

## Survey

### Information from Autistic Girls

#### Camouflaging Autistic Traits Questionnaire (CAT-Q)

The CAT-Q is a 25-item self-report questionnaire to measure strategies and behaviors used to camouflage autistic traits (Hull et al., 2019). Autistic girls received a modified version of the CAT-Q (Halsall et al., 2021) in which items appeared on a 4-point Likert scale and reverse-valance items (*n* = 4) eliminated. This modified version of the CAT-Q had a Cronbach’s alpha of 0.88. We then summed items to obtain a total CAT-Q score (higher = greater camouflaging).

#### Specific Aspects of School Camouflaging

Respondents answered questions about their camouflaging experiences related to the inclusive school context. Given that no well-developed camouflaging measures exist specific to this setting, questions were developed based on current literature on camouflaging and the experiences of autistic females in schools. See Table 2.

**Table 2:** Closed-Ended Items for School-Specific Camouflaging Questions

Topic	Description	Matrix item names	Response options	Alpha
Settings	Matrix of items examining extent in which girls camouflaged across different school contexts	General education classroom; Special education/resource room^a^; Hallway; Cafeteria; Physical education^a^; Fun elective with peers (e.g., theater, music, art, etc.); Other activities with peers (e.g., study hall, busing, etc.); Home^b^	(1) Never (0 days per week) to (4) Always (5 days per week); “Not applicable” also an option	.82
People	Matrix of items examining extent in which girls camouflaged across different people in school	Teacher feel close to; Teacher don’t know as well; Paraprofessional/teacher assistant^a^; Related service providers^a^; Typically developing friends; Friends with autism or another disability; Typically developing peers do not feel close to; Peers with disabilities do not feel close to; Romantic interest^a^; Parents	(1) Never (0 days per week) to (4) Always (5 days per week); “Not applicable” also an option	.73
Time started camouflaging	Single item examining when girls recall starting to camouflage	NA	(1) As early as I can remember to (4) High school; “I don’t know” also an option	NA
Duration of camouflaging	Single item examining how long girls camouflage in school	NA	(1) Never to (4) All day in school	NA
Perceived success	Single item examining how successful girls perceive their camouflaging	NA	(1) Not at all successful to (4) Very successful	NA
Parent familiarity	Single item examining how aware girls perceived their mothers to be in their daily experiences in school	NA	(1) Unaware to (4) Very aware	
Benefits	Matrix of items examining perceived benefits of camouflaging	Easier to make friends; Easier to work with classmates; More attractive to romantic interest^a^; Look smarter to others; Less awkward when talking to others	(1) Never (0 days per week) to (4) Always (5 days per week	.58^c^
Costs	Matrix of items examining perceived costs of camouflaging	Exhausted; Anxious; Depressed; Meltdowns; Identity loss	(1) Never (0 days per week) to (4) Always (5 days per week	.71
School supports	Matrix of items examining girls’ perceptions of how different school supports would reduce the pressure to camouflage	Adult awareness of autism; Peer awareness of autism; More time alone to recharge; Teachers providing clear expectations and structure; Teachers checking for understanding; A person to talk about emotions; Less reliance on paraprofessional/teacher assistant^a^; Hearing good things about autism from others; Social skills instruction; Structured opportunities to make friends	(1) Would not help to (4) Would be extremely helpful	.73
General disclosure	Single item where participants rated agreement with following statement: “I am generally open about acknowledging and discussing my status as an autistic person” (Cage & Troxell-Whitman, 2020)	NA	(1) Strongly disagree to (4) Strongly agree	NA
Disclosure to others	Matrix of items examining extent girls disclosed their autism to different people	Typically developing friends; Friends with autism or another disability; Typically developing peers do not feel close to; Peers with disabilities do not feel close to; Romantic interest^a^	(1) Never (disclose to no one) to (4) Always (disclose to all)	.72
Negative peer experiences	Matrix of items examining the extent to which girls experience bullying and exclusion in school	Bullying by peers; Bullying by teachers; Exclusion by peers; Exclusion by teachers; Loneliness	(1) Never (0 days per week) to (4) Always (5 days per week	.76

^a^ = item deleted. ^b^ = location where participant lived, including with caregivers, siblings, etc. ^c^ = items individually correlated with other camouflaging and background variables

Settings, people, benefits, costs, and school supports were examined via questions arrayed on a matrix (i.e., with row items having identical column response options). Besides camouflaging-specific questions, respondents also answered questions related to autism disclosure across different people in school and negative social experiences. As over half of respondents did not take physical education or attend a special education/resource room—and over one-third did not have a paraprofessional, related service provider, or romantic interest—these variables were dropped. For the remaining items, Cronbach’s alphas were calculated, and an average domain score was obtained (sum divided by number of items) for each matrix. Time started camouflaging, duration of camouflaging, perceived success of camouflaging, parent familiarity of school day, and general autism disclosure were each examined via a single item.

#### Open-ended Responses

Participants answered three open-ended questions: (1) What advice would you give to teachers and other adults in school to make autistic girls feel more included?; (2) What advice would you give to other autistic girls?; and (3) Is there anything else you’d like to share about your experiences camouflaging in school?

### Information from Mothers

#### Demographics

For demographics about themselves and their families, mothers reported on their race/ethnicity, marital status, education, income, employment status, number of typically developing and neurotypical children, autistic daughters’ diagnostic status, and autism organization/group membership. Mothers also answered the 36-item Broad Autism Phenotype Questionnaire (BAPQ, Hurley et al., 2007; for this study, alpha = 0.95).

#### Background

Mothers also reported about their autistic daughters, including the daughter’s age, diagnosis, age of diagnosis, co-occurring conditions, provider who diagnosed daughter, psychotropic medicine use, portion of time in inclusion, when child was first included, grade, and presence of 504 or IEP plan. Mothers also answered about their daughters’ social and behavioral challenges over the past 6 months via the Social Responsiveness Scale-2 (SRS; Constantino & Gruber, 2012; alpha = 0.94). Other measures included a checklist of their child’s current diagnoses of anxiety, depression, ADHD, or other, as well as the Revised Children’s Anxiety and Depression Scale – (RCADs; Ebesutani et al., 2017; Sterling et al., 2015) – parent form (alpha = 0.92). Via a separate Zoom interview, mothers answered CAT-Q questions (mothers’ perceptions of daughters’ camouflaging behaviors), as well as aspects of school camouflaging, disclosure, bullying/exclusion, and open-ended responses.

### Analyses

Four sets of analyses were conducted. Given the numbers of independent analyses and relatively small sample size, results were considered significant at *p* < 0.01. Prior to analyses, all numerical variables were checked for normality.

### Nature and Extent of Daughters’ Camouflaging

To determine whether items showed different mean values, girls’ scores on CAT-Q items were compared with a one-way repeated-measures ANOVA. Post-hoc analyses identified which items were higher or lower relative to the Grand Mean (Silverstein, 1975). To examine reliability, Cronbach’s alphas were examined. Afterward, daughters’ scores on individual items of the CAT-Q were summed into a composite camouflaging score. Similarly, for aspects of school camouflaging (e.g., settings camouflaged in, people camouflaged to), one-way repeated measures ANOVAs were conducted to examine whether individual items showed different mean values. Domain scores were then correlated with settings, people, benefits, costs, and school supports.

### Connections Across Camouflaging Domains and of Domains to Individual Characteristics

To examine how personal characteristics related to camouflaging, Pearson *r* correlations were conducted between items on a numerical scale (e.g., age of diagnosis, SRS-2) with daughters’ CAT-Q and domain scores for aspects of camouflaging (i.e., settings, people, pros, cons, school supports). For individual characteristics that were part of a scale (i.e., SRS-2, RCADs), individual scale items in need of reverse scoring were recoded. All scale items were then computed into a single variable. Cronbach’s alphas were also calculated to assess reliability. For items on a categorical scale, independent *t*-tests (for variables with 2 groups) and one-way ANOVAs (3 or more groups) were conducted with daughters’ CAT-Q scores.

### Open-Ended Responses

All interviews were transcribed verbatim by research assistants, de-identified, and imported into Dedoose (Version 9.0.90). Using constant comparison, existing codes were compared with previous uses to ensure consistency (Strauss & Corbin, 2008). Using parallel coding (Thomas, 2006), transcripts were independently open coded by two coders. To prioritize participants’ perspectives, we used in vivo coding, assigning codes using participants’ actual language (Saldaña, 2013). Coded responses ranged from one-two sentences to a paragraph. Coders then met for consensus. Axial coding was then used to identify themes and develop code names with corresponding definitions. Finally, full transcripts were revisited by using these definitions to assign codes to applicable responses.

To support the credibility and trustworthiness of data, an audit trail was established to document raw data (e.g., interview dates and times, transcripts) and memos from all coding steps. Bias was reduced by using a team-based approach and involving a third (outside) member to serve as a peer debriefer. We also member-checked themes with a dyad, including an autistic mom. Finally, we conducted negative case analysis to look for any disconfirming cases in identified themes.

### Integrating Quantitative and Qualitative Data

Quantitative and qualitative data were collected concurrently and analyzed separately. We then integrated quantitative and qualitative data through *merging* (i.e., bringing data together for analysis) in a joint display figure to facilitate interpretation (Fetters et al., 2013).

### Community Involvement Statement

This study was conducted by a neurodiverse research team. Two members were autistic and two were non-autistic. Both autistic members had extensive life experiences camouflaging. One was involved in the survey design and interviewed participants, with both autistic authors leading the first round of coding (which was verified with non-autistic peer debriefers). Given the “double empathy problem,” having a neurodiverse team enabled us to empathize with the lived experiences of autistic people (Milton, 2012).

## Results

### Nature and Extent of Daughters’ Camouflaging

All autistic females reported camouflaging. Most girls (67.7%, *n* = 21) reported camouflaging most or all of the school day and perceived themselves to be “mostly successful” in hiding their autistic characteristics (54.8%, *n* = 17). Over one-half (*n* = 19) felt their mothers were “aware” or “very aware” of their daily experiences (only 6.5% felt their mothers were unaware). Girls varied with regards to the time they started camouflaging, with one-fourth (25.8%; *n* = 8) of participants each reporting, “I don’t know,” elementary school, and middle school. Daughters’ camouflaging behaviors differed across CAT-Q scale items, with a large effect size, *F*(20, 600) = 11.67, *p* < 0.001, η_p_^2^ = 0.28. Girls behaved differently at school versus home and considered the impression that others had of them. Girls rated lower copying what other people wore.

In their own answers, girls reported themselves differentially camouflaging across school settings, *F*(5, 145) = 14.09, *p* < 0.001, η_p_^2^ = 0.33 and persons, *F*(3.42, 102.53) = 28.14, *p* < 0.001, η_p_^2^ = 0.48, Greenhouse–Geisser correction. Girls camouflaged most in general education classes and least at home; most with neurotypical peers that they did not know well, least with neurodivergent friends or parents. Camouflaging costs also differed across items, *F*(4, 116) = 7.09, *p* < 0.001, η_p_^2^ = 0.20, with exhaustion the highest and identity loss the lowest.

Finally, girls’ ratings differed across school supports, *F*(8, 240) = 5.01, *p* < 0.001, η_p_^2^ = 0.14. Girls rated highest having more adult autism awareness and having time alone to recharge; lowest ratings went to having an adult in school to talk about emotions and social skills classes. Most girls (87.1%; 27) reported it would help or be extremely helpful to have adult awareness of autism/camouflaging and time alone to recharge; only 48.4% (15) rated as helpful-extremely helpful social skills classes and having an adult to talk about emotions, McNemar’s *p’s* < 0.01 comparing high vs. low items.

### Ties Across Camouflaging Domains and of Domains to Individual Characteristics

Girls who camouflaged more and perceived themselves successful at it were more likely to camouflage across settings and people (*r’s* from 0.52 to 0.82, *p*’s < 0.01). Further, camouflaging settings, costs, and people interrelated (*r’s* from 0.53 to 0.77, *p’s* < 0.001). Camouflaging costs were positively associated with CAT-Q scores, *r*(28) = 0.70, *p* < 0.001. The school support domain showed no relations to any aspects of camouflaging.

The main individual characteristics relating to camouflaging were the age at which a girl was diagnosed and time since participants’ autism diagnosis. The costs of camouflaging were positively correlated with the girls’ age of diagnosis,* r*(28) = 0.59, *p* < 0.001, and, negatively, with time since diagnosis, *r*(28) = −0.54, *p* < 0.01. Comparing those diagnosed earliest (≤ 5 years; *n* = 7) vs. latest (≥ 14 years, *n* = 14), CAT-Q scores were lower for those diagnosed earliest (*m* = 41.71; *SD* = 7.52) versus latest (*m* = 54.43, *SD* = 9.78); *t*(19) = −3.01, *p* < 0.01. Camouflaging costs were also lower in girls diagnosed earliest (*m* = 1.80; *SD* = 0.57) versus latest (*m* = 2.68; *SD* = 0.58), *t*(18) = −3.25, *p* < 0.01. No other findings were noted.

Only a few correlations emerged regarding camouflaging’s benefits. Specifically, looking “smarter” to others correlated with number of settings camouflaged, *r*(28) = 0.54, *p* < 0.01, and CAT-Q scores* r*(29) = 0.51,* p* < . 01. Looking less “awkward” related to both camouflaging costs *r*(28) = 0.55, *p* < .01 and CAT-Q scores *r*(29) = 0.56, *p* < 0.01. Mothers’ BAPQ scores were unrelated to daughters’ camouflaging on the CAT-Q or domains.

### Four Themes Derived from Interviews

#### Autistic Identity

Mentioned by 38.7% (12) of these girls, participants considered autism a fundamental part of their identities, involving both strengths and challenges. As one participant stated, autism is “part of who I am.” Many girls “always knew” that they were “different” before receiving a diagnosis, even as many reported receiving little support in understanding their autism. One girl explained:

“…I sat alone in the dark up all night wondering why I didn’t get to just know all these things people seemed to have been born with. I learned it because I had no choice. I had to first be broken down to be able to rebuild myself.”

Finally, many girls described the balance between camouflaging and maintaining their autistic identity. They did not feel “normal” enough to have meaningful social relationships with general education peers, yet “not autistic enough” to be embraced by the autism community.

#### Negative Peer Experiences

This theme (35.5%; 11) included experiencing bullying and exclusion, especially during middle school. Many girls reported having few friends and feeling condescended to by peers. As one girl stated, “They think I’m stupid.” Girls also reported that peers in general education did not know how to interact with them. They often were the last person picked for group projects or teams during gym, further exacerbating their feelings of isolation.

#### Negative Consequences of Camouflaging

This theme, mentioned by 32.3% (10) of the sample, concerned how camouflaging feels and its adverse consequences. As one girl noted,

“It’s not a very sharp pain... if we’re thinking about when you get hurt, it’s not like, ‘oh, a shard of glass has entered my arm.’ It’s not like my best friend told me they don’t want to be friends with me anymore…it’s way more sort of numb and I think of it as round, like a bruising sort of pain…it just sort of coats over you and so you aren’t very aware from day to day of like, ‘oh my God, this is so hard’…you’re a bit like, ‘it’s not that big.’ But really, it’s huge.”

Participants described camouflaging as largely negative, yet a behavior that they felt continued pressure to engage in and “obsessed” about during school. Many girls reported that, throughout their lives, they received feedback that there was something “off” about them. They thus spent a large portion of their school day camouflaging.

#### Value of Neurodivergent Friends

Of those girls with quality friendships, many (19.4%; 6) reported friendships with peers who were also autistic and/or neurodiverse, including males. Participants felt these neurodiverse peers understood them better, enabled them to have conversations about their autism, had similar interests, spoke more directly, and helped them navigate a neurotypical world. As one participant remarked, “If I didn’t have my neurodivergent friends, I don’t think I would have been able to get through the school year.”

### Comparison of Interview Themes to Quantitative Findings

**Fig. 1:**
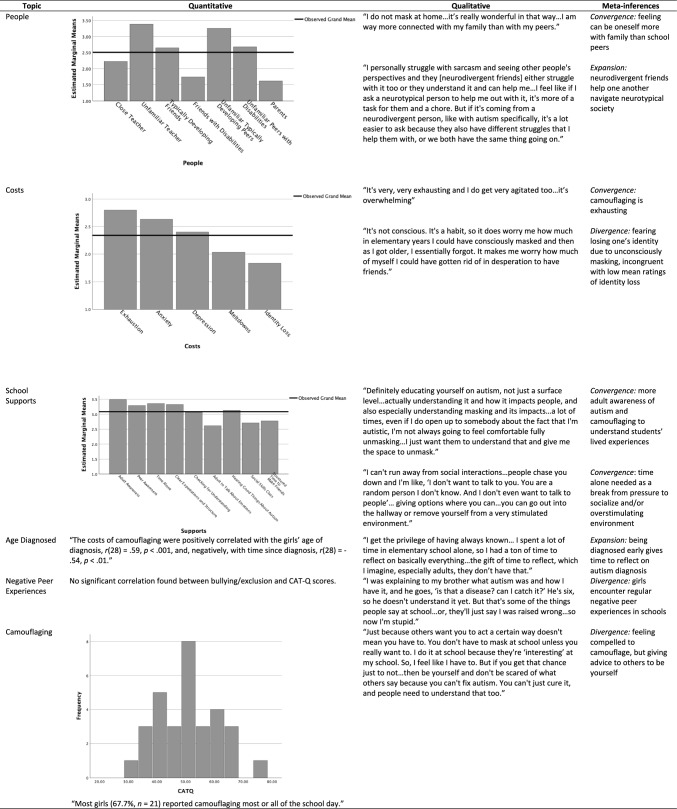
Joint Display of Quantitative, Qualitative, and Mixed Method Inferences

When comparing girls’ ratings to their open-ended responses, both commonalities and discrepancies occurred (see Fig. 1). In line with quantitative ratings, girls described how they do not camouflage at home, find camouflaging exhausting, and desire breaks from overstimulating school environments. But in other cases, the two types of data diverged. For example, girls rated experiencing identity loss the least; however, one girl elaborated that she fears how much of herself she might have lost from years of unconsciously masking. Similarly, while bullying/exclusion was uncorrelated with camouflaging, girls described numerous instances of negative peer interactions. Finally, while over half of girls rated camouflaging most of the school day, they still advised other autistic girls to “be yourself” and “never forget who you are.”

Finally, open-ended answers sometimes expanded on quantitative data. In interviews, for example, girls noted how neurodivergent friends enabled each partner to navigate a neurotypical world. In line with ties of earlier diagnosis and lesser degrees of camouflaging, one girl noted that being diagnosed early was a “privilege” because it gave her the “gift of time to reflect.”

## Discussion

Autistic individuals experience varying degrees of intensity and levels of needed supports, including school-aged autistic females. To date, however, autistic girls have been under-examined in research and practice. This study is among the first to examine autistic females’ experiences camouflaging quantitatively and qualitatively in inclusive school contexts. This study has four main findings, each with implications for both research and practice.

Our first finding concerned the nature and extent of camouflaging. All girls mentioned that they camouflaged to some extent, with most indicating they camouflaged “sometimes” or more during the school day. Autistic females camouflaged more in general education classes with neurotypical peers, least at home. They also camouflaged most to teachers and typically developing peers that they did not know as well, least to friends with disabilities and parents. After camouflaging in school, participants also reported feeling exhausted and anxious. Finally, girls reported that schools could help most by increasing the autism awareness of school personnel and by allowing girls to have time alone; the least helpful school supports involved having an adult to talk about emotions and social skills classes. Girls’ lower rating of social skills classes is noteworthy, especially given that many interventions for autistic people focus on social skills training (Wong et al., 2015).

Our second finding concerned relations among camouflaging’s various aspects, with the domains of settings, people, and costs all strongly correlated (benefits less so). If girls camouflaged more across settings, they also camouflaged more to a greater variety of people. Girls reported that camouflaging enabled them to look “smarter” and less “awkward” when interacting with others. Autistic girls might thus perceive the benefits from camouflaging to be worth the mental costs. The adult literature suggests that camouflaging enables individuals to receive an education, maintain a job, and establish relationships with neurotypical people (Hull et al., 2017). Similarly, autistic females often felt that camouflaging was essential to navigate a school environment that would otherwise be inhospitable. The main personal characteristic associated with camouflaging was age of diagnosis. Those girls diagnosed earlier reported lower camouflaging costs and lower overall camouflaging scores. In line with earlier studies (Milner et al., 2023), autistic females displayed more camouflaging when diagnosed at later ages (i.e., $\ge$ 14 years).

Our third finding identified four themes. Girls spoke of how autism is part of their identity, in line with recent research indicating that autism is both a social identity and a clinical diagnosis (Cooper et al., 2021). These girls also noted how it impacts them, further elaborating on the costs of camouflaging, yet less often elaborating on its benefits. Consistent with Greenlee et al. (2020) and Halsall et al. (2021), girls reported numerous negative peer experiences, primarily related to relational aggression and exclusion. The last theme addressed the value of neurodivergent friends, particularly autistic friends.

Juxtaposing qualitative and quantitative data, our last finding revealed both convergence and divergence between closed- and open-ended questions. In line with quantitative ratings, most girls described home as their haven and that they camouflage less with family. Conversely, several girls reported that they dreaded going to school because it required them to “perform.” Likewise, research in social identity suggests that individuals with disabilities often face stigma, a discrediting label that can alter the way in which others perceive them; to conceal their disability, individuals might thus “perform” for others in efforts to conceal their disability and benefit from the privileges afforded to the dominant group (Goffman, 1963). Like quantitative findings indicating less camouflaging with neurodivergent friends, many girls elaborated that the presence of neurodivergent friends, including autistic friends, made school safer and more tolerable.

But quantitative and qualitative findings also sometimes diverged. For example, while these autistic girls rated feelings of identity loss the lowest, in interviews autistic identity emerged as a major theme. Similarly, whereas autistic girls rated lowest feelings of identity loss, in interviews girls talked about the conflicts they experience between being neither “normal enough” nor “autistic enough.” Straddling this line often resulted in increased feelings of isolation. For some girls, their autistic identity acted as a buffer against feelings of identity loss. Cooper et al. (2023) also found that higher autism satisfaction in young people was associated with greater psychological well-being. Finally, although all girls reported camouflaging at school, many advised other autistic females to be themselves. Girls seemed to be striking a balance between feeling obligated to camouflage but recognizing its negative costs.

### Implications for Research

These findings have implications for both research and practice. Notably, little is known about how camouflaging changes over childhood. Although this study was cross-sectional, some participants reported a long history of camouflaging in schools, with several camouflaging as early as they could remember. Overall, one-fourth of girls reported that they started camouflaging in middle school, and several mothers reporting that middle school was a particularly tough developmental period. More longitudinal studies are needed to explore how camouflaging in school changes over time.

Additionally, although many girls were succeeding academically, many had diagnosed anxiety or depression and were taking psychotropic medications. More research is also needed to better understand the relationship between camouflaging and internalizing psychopathologies. Similarly, more research is needed on what schools can do to better support autistic females’ psychological well-being.

In addition, studies are needed to explore to what extent findings are specific to autistic females in general education school settings for most of the school day. We need to understand, for example, how the perspectives of these girls compare to those of girls with more extensive support needs and/or co-occurring intellectual disability (ID). Students with more significant cognitive disabilities are sometimes included in general education classrooms (National Center for Education Statistics, 2023). Of the few studies that even examines autistic females’ educational experiences, most are comprised of participants without ID (Goscicki, 2023). Further, more research is needed to explore to what extent non-autistic girls (both neurotypical and neurodivergent, including ADHD) engage in camouflaging behaviors, as well as their respective costs and benefits. For example, in the adult literature, adults of varying neurotypes engaged in camouflaging behaviors and experienced negative impacts, although some behaviors (e.g., sensory suppression) were more specific to autistic people (Miller et al., 2021). More research is also needed to explore how camouflaging in school varies by sex and gender identity.

Finally, future research should examine whether distinctions exist between camouflaging and codeswitching, “the practice typical of individuals proficient in two or more registers, dialects, or languages who will switch from one to the other depending on the conversational context or in order to enhance linguistic or social meaning” (APA Dictionary of Psychology, 2018). While codeswitching has been discussed in other areas of intersectionality (e.g., race), recent conversations have focused on whether practitioners should support autistic individuals who wish to present themselves in a neurotypical manner to participate more equitably in certain contexts (Gerlach-Houck & DeThorne, 2023). Within the neurodiversity community, the terms “codeswitching” and “masking” have also been used interchangeably (Price, 2022). Future research should therefore examine the extent to which individual autistic girls desire to codeswitch, how camouflaging and codeswitching are similar or different, and, if some autistic girls are interested, how best these girls can codeswitch to achieve maximum benefits for themselves in their everyday lives.

### Implications for Practice

These findings also have practical implications. Many girls felt like their teachers did not know how to support their needs, which heightened feelings of isolation and their feeling different from peers. Girls reported that the most helpful school support would be increased teacher knowledge of autism (including camouflaging). School practitioners should also empower students to explore, identify, and communicate their autistic needs. Given the diversity within the autism community, each student is likely to have unique needs that may change over time. Besides acquiring more knowledge of autism, practitioners would be wise to get to know their students at an individual, personal level to individualize supports.

Another helpful school support would be time alone to recharge. Given that schools require social interaction throughout the day, autistic females’ bandwidths might be limited. Personnel should be careful not to pressure students to socialize if the student does not want to. Schools might consider offering autistic females breaks or leisure time throughout the school day, along with a quiet room or other designated physical location to do so. Further, school personnel should normalize the value of alone time, so that these girls do not feel ostracized for tending to their needs to recharge.

### Limitations

Although this study focused exclusively on autistic females, incorporating the perspectives of non-autistic adolescents would have pinpointed how much these findings were particular to autistic females. This study was also limited to mostly girls who were White, middle-class, and of well-educated, married parents. Further, our study may have had low power from our small sample size and modifications to the CAT-Q, and the large numbers of separate analyses may have resulted in Type I and/or Type II errors. We also did not examine diagnosis levels of autism or intersections of different gender identities and camouflaging, even as the autism community is comprised of greater gender diversity, including individuals identifying as non-binary or transgender (Corbett et al., 2023). Additionally, given that social communication deficits are a hallmark of autism, interviewing daughters comes with inherent challenges, though we sought to address these concerns through accessible methods. Finally, one must acknowledge that the girls themselves could have been camouflaging to researchers.

## Conclusion

Despite their placements alongside general education peers, autistic females do not feel socially included. Until recently, most research has focused on getting autistic individuals to change their behaviors to fit in; schools now need to better support autistic students and normalize neurodiversity. But beyond normalizing, educators should encourage students to self-advocate their autistic needs and become allies for those students who cannot yet advocate for themselves. By validating neurodiversity in schools, autistic females might feel less pressure to camouflage. Ultimately, we need to develop interventions and cultures that appreciate instead of stigmatizing or simply tolerating differences.
